# The effect of McInnes solution on enamel and the effect of Tooth mousse on bleached enamel: An *in vitro* study

**DOI:** 10.4103/0972-0707.44058

**Published:** 2008

**Authors:** H E Darshan, N D Shashikiran

**Affiliations:** Department of Pedodontics, JSS Dental College and Hospital, Mysore-570 015, India; 1 Department of Pedodontics, College of Dental Sciences, Davangere-577 004, India

**Keywords:** Amorphous calcium phosphate, bleaching, casein phosphopeptides, enamel, hardness, remineralization, Vickers hardness number

## Abstract

**Aims:**

To evaluate the effect of McInnes bleaching agent on the micro hardness of enamel before and after bleaching and to evaluate the effect of G C Tooth Mousse on the bleached enamel surface for its microhardness.

**Materials and Methods:**

McInnes bleaching solution, Casein phosphopeptide-amorphous calcium phosphate CCP-ACP (G C Tooth mousse) artificial saliva (Dept of Oral Pathology, College of Dental Sciences, Davengere), deionized water, Vickers Micro Hardness tester (Zwick/ZHV, Germany), freshly extracted teeth, cold cure acrylic, Diamond disc (Horico - PFINGST New jersey USA, KAVO- Germany), straight handpiece (kavo peca reta) and plastic moulds (6.5 × 2 mm). The purpose of this study was to evaluate and compare microhardness of the sound enamel surface by Vickers Hardness Number before and after bleaching with McInnes solution, and to evaluate the effect of casein phosphopeptide amorphous calcium phosphate (G C Tooth Mousse) on the bleached enamel surface for its microhardness.

**Statistical analysis:**

The data obtained from the test were subjected for statistical analysis and are presented as range, mean and standard deviation. P value of 0.05 or less was considered for statistical significance. The changes in microhardness at different times of assessment were analyzed using the paired ‘t’ test

**Results:**

All the samples showed decrease in the microhardness after two cycles of bleaching, though immediately after bleaching the decrease in the microhardness was not significant (P = 0.34). However, after the second cycles, it showed a significant decrease (P<0.01) in the microhardness. After application of remineralization solution (GC Tooth mousse), the samples showed a marginal increase in the microhardness (P<0.05) after seven days and a marked increase after fourteen days (P<0.001).

**Conclusion:**

McInnes bleaching agent does decrease the microhardness of enamel by causing enamel demineralization and GC Tooth mousse used in the study causes an increase in the microhardness of bleached enamel by maintaining a high gradient of calcium and phosphate ions at the enamel subsurface.

## INTRODUCTION

Tooth enamel is the most mineralized tissue of the human body. Its composition is 96 wt% inorganic material, 4 wt% organic material and water. Human teeth are exposed to a different point to point pressure during mastication. Therefore, an understanding of the masticatory strain distribution throughout the tooth and an understanding of the fact that the prediction of stresses and strains are altered by dental restorative procedures, age and disease are important.[1]

The technique of bleaching or whitening teeth was first described in 1877.[2] It was in the year 1916 that Dr. Walter Kane used hydrochloric acid to successfully remove the fluorosis stains. In the year 1937 Ames reported an alternative for removing fluorosis using hydrogen peroxide instead of hydrochloric acid. Later it was McInnes who reported a technique where hydrogen peroxide, hydrochloric acid and ethyl ether were used. This technique has been found to be successful for bleaching the teeth of patients with endemic fluorosis.[3]

Increased frequency of acid exposure in bleaching tends to alter the total demineralization/ remineralization amounts, resulting in significantly greater amounts of mineral loss.[4] The presence of fluoride acts as a remineralizing agent, by forming a calcium fluoride layer.[5]

Casein phosphopeptide-amorphous calcium phosphate (CPP-ACP) nanocomplexes, which have been shown to prevent demineralization and promote remineralization of enamel subsurface lesions in *in situ* caries models.[6] The CPP, by stabilizing calcium phosphate in a solution, maintains high-concentration of gradients of calcium and phosphate ions into the subsurface lesion and thus affects high rates of enamel remineralization.[7]

Various methods have been used to analyze tooth demineralization and remineralization when acidic bleaching is used, including techniques ranging from direct measures of mineral gain/ loss as in microradiography to indirect measures like iodide permeability and surface microhardness.[8]

Based on these observations the present study was undertaken at College Of Dental Sciences, Davengere, to investigate the effect of McInnes bleaching on enamel microhardness and subsequent remineralization by CCP-ACP on bleached enamel.

## MATERIALS AND METHODS

The present study was conducted in the Department of Pedodontics and Preventive Dentistry, Collage of Dental Sciences, Davangere, in collaboration with the Department of Metallurgy, Indian Institute Of Science, Bangalore.

### Materials used

1. McInnes Bleaching solution
2. CCP-ACP(G C Tooth mousse)
3. Artificial saliva (Dept of Oral Pathology, College of Dental Sciences, Davengere)
4. Deionized water

### Equipment used for testing

1. Vickers Micro Hardness tester (Zwick/ZHV, Germany)

### Materials used for preparation of samples

- Freshly extracted teeth
- Diamond disc (Horico – horico- PFINGST New jersey USA)
- Straight handpiece (kavo peca reta)
- Plastic moulds(6.5 × 2mm)
- Cold cure acrylic (DPI)

### Materials used for preparing artificial saliva

- Calcium chloride 0.22g/l
- Sodium phosphate 1.07g/l
- Sodium bicarbonate 1.68/l
- Sodium azide 2g/l
- Distilled water one liter

### Materials used for remineralization

GC tooth mousse (Recaldent) which contains: CPP-ACP, Glycerol, Silicon dioxide, Zinc oxide, cmc-na, propylene glycol, titanium dioxide, phosphoric acid, guargum, sodium saccarien, ethyl p-hydroxybenzoate, butyl p-hydroxybezoate, propyl p-hydroxybenzoate, Magnesium oxide, D Sorbitol, Xylitol and Pure water

### Inclusion criteria

Only permanent non carious teeth that were indicated for extraction were included in this study.

### Preparation of specimens

Freshly extracted teeth were selected. The teeth were cut sagittally, using diamond disc (Horico – horico- PFINGST New jersey USA) and the buccal surface were impregnated in the cold cure acrylic resin facing upwards. Using the plastic moulds the resins were made into pellets (6.5mm × 2mm thickness). A total of ten specimens were made, which were then kept in artificial saliva to prevent dehydration.[8] The samples were rinsed in water and dabbed dry with absorbent paper before subjecting them for baseline hardness test.[9]

### Procedure for microhardness test

For microhardness testing the “Zwick Roell Indentec” (Zwick, Inc, Germany) was used for measuring Vickers hardness test. The tests were carried out according to the manufacturer's instructions.[10] The test specimens were placed on the stage of the tester and stabilized. Then area to indent was selected by focusing with 10× objective lens. After this, the test was carried out where the indentations were made with a rate of 100g load for 30 seconds, never close to any edge of the specimen. The indentation formed was viewed and measured on the display monitor with 10× objective lens. The average microhardness of the specimen was determined from two indentations to avoid any discrepancy, since the enamel surface has a curvature. The procedure was repeated for all the ten specimens.

### Preparation of bleaching agent

McInnes bleaching solution consists of a mixture of 1ml of 36 % hydrochloric acid, 1ml of 30 % hydrogen peroxide and 0.2 ml of anesthetic ether which is mixed in the ratio of 5:5:1. The mixture is prepared freshly in a dappen dish before each application.[11]

The bleaching agent thus prepared bleaching agent was applied to the enamel surface using a cotton applicator for five minutes (first cycle of bleaching). It was then washed under deionized water, damped dry with absorbent paper and then subjected for microhardness test. After this, the samples were stored in artificial saliva for 24 hours to prevent dehydration. Again after 24 hours the second application of bleaching agent was carried out as described earlier and the microhardness values were recorded. Then the samples were applied with remineralizing agent (GC Tooth mousse) for fourteen days (second cycle of remineralization).[12,13,14]

### Application of GC tooth mousse

GC- Tokyo Tooth mousse (Recaldent) was applied with cotton applicator tips on the post bleached samples, everyday for seven days with minimum application time of three minutes. The samples were then washed under deionized water, stored in artificial saliva for seven days (first cycle of remineralization) after which the samples were tested for microhardness and the values were recorded as described earlier. Following this, GC Tooth mousse was applied for seven more days and at the end of fourteen days (second cycle of remineralization) the samples were subjected for microhardness testing using the same procedure as described earlier.

The recorded values are subjected to statistical analysis.

### Results

The data obtained from the following test were subjected for statistical analysis and are presented as range, mean and standard deviation [Table 1]. A “ *P* ” value of 0.05 or less was considered for statistical significance. The changes in microhardness at different times of assessment were analyzed using the paired “t” test

**Table 1:** Mean, *P* value and percentage changes of microhardness from baseline

	1^st^ Cycle of bleaching	2^nd^ Cycle of bleaching	1^st^ Cycle of remineralization	2^nd^ Cycle of remineralization
Mean ± SD	296.2 ± 17.3	290 ± 16.6	304.3 ± 18.5	307 ± 17.4
P value	= 0.034 NS	<0.001 HS	<0.05 S	<0.05 S
Percentage changes from baseline 297.2 ± 17.6	0.3% Decrease	2.3% Decrease	2.2% Increase	3% Increase

SD = Standard deviation; S= Significant; HS= Highly significant

[Table 1] shows the mean, *P* value and percentage changes of microhardness from the baseline. The first comparison was between the baseline and the first cycle of bleaching. The mean and standard deviation at baseline was 297.2 ± 17.6 and for the first cycle of bleaching was 296.2 ± 17.3. When these values were compared it showed a decrease by a small margin of 0.3%, which was not significant (*P* = 0.034). The next comparison was between the baseline and the second cycles of bleaching carried out 24 hours later. The mean and standard deviation after the second cycle of bleaching was M=290 SD= 16.6, which showed decrease by 2.3% and was highly significant (*P* < 0.001). The third comparison was between the baseline and the first cycle of remineralization (M=304.3, SD=18.5), which showed increase by 2.2% and was significant (*P* value < 0.05). The fourth comparison was between the baseline and the second cycle of remineralization (M=307.0 ± 17.4) which showed 3% increase from the baseline values which was significant (*P* < 0.05).

[Table 2] shows the mean, *P* value and percentage changes of microhardness from the first cycle of bleaching. The values obtained after first cycle of bleaching (M=296.2, SD= 17.3) were compared with the values of the second cycle of bleaching (M= 290.0, SD=16.6) which showed 2% decrease and was significant (*P* < 0.05). The next step was to compare the values of first cycle of bleaching with first cycle remineralization (M=304.3, SD= 18.5) which showed 2.5% increase in Vickers's hardness number (VHN), and was significant (*P* < 0.01). When the first cycle of bleaching was compared with 2^nd^ cycle of remineralization (M= 307, SD = 17.4), it showed an increase by 3.24% and was significant (*P* < 0.01).

**Table 2:** Mean, *P* value and percentage changes of microhardness from the 1^st^ cycle of bleaching

	2^nd^ cycle of bleaching	1^st^ cycle of remineralization	2^nd^ cycle of remineralization
Mean ± SD	290 ± 16.6	304 ±.3 18.5	307 ± 17.4
*P* value	P<0.05 S	P<0.01 S	P<0.01 S
Percentage change from first cycle of bleaching 296.2 ± 17.3	2% decrease	2.5% increase	3.24% increase

S= Significant; SD= Standard deviation

[Table 3] shows the mean, *P* value and percentage changes of microhardness changes between first cycle of remineralization and the second cycle of remineralization. The comparison between the first cycle of remineralization (M=304.3 ± SD= 18.5) and the second cycle of remineralization (M=307 ± SD 17.4) showed increase in the VHN by 1% and was significant (*P* < 0.05)

**Table 3:** Mean, *P* value and percentage changes of microhardness from the 1^st^ cycle of remineralization

	2^nd^ cycle of remineralization
Mean ± SD	307 ±17.4
*P* value	<0.05 S
Percentage change from first cycle of remineralization 304.3 ± 18.5	1%

S= Significant; SD= Standard deviation.

**Figure 1:**
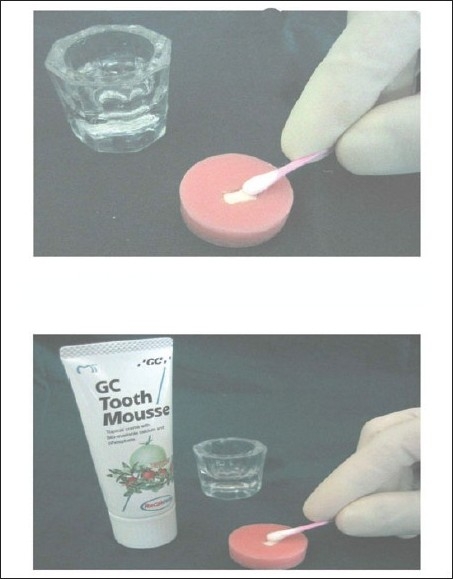
Application of bleaching agent and application of GC tooth mousse

**Figure 2:**
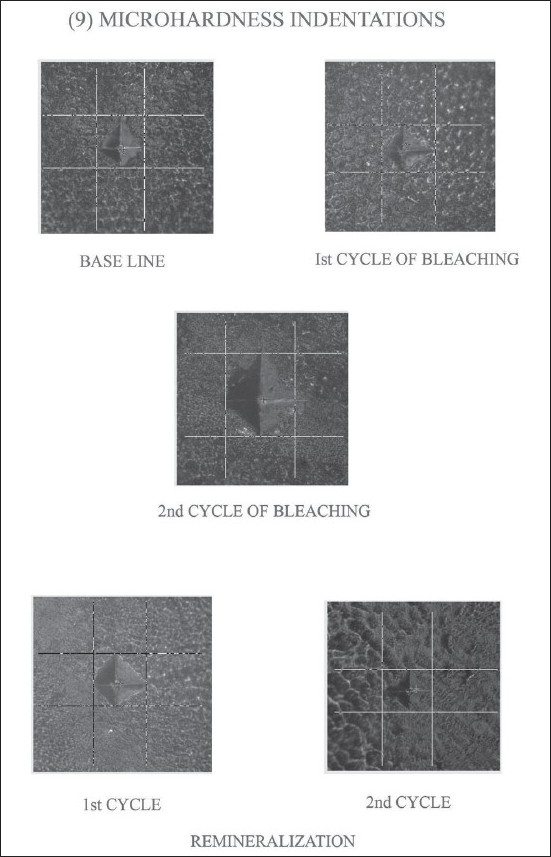
Micro hardness Indentations

**Figure 3:**
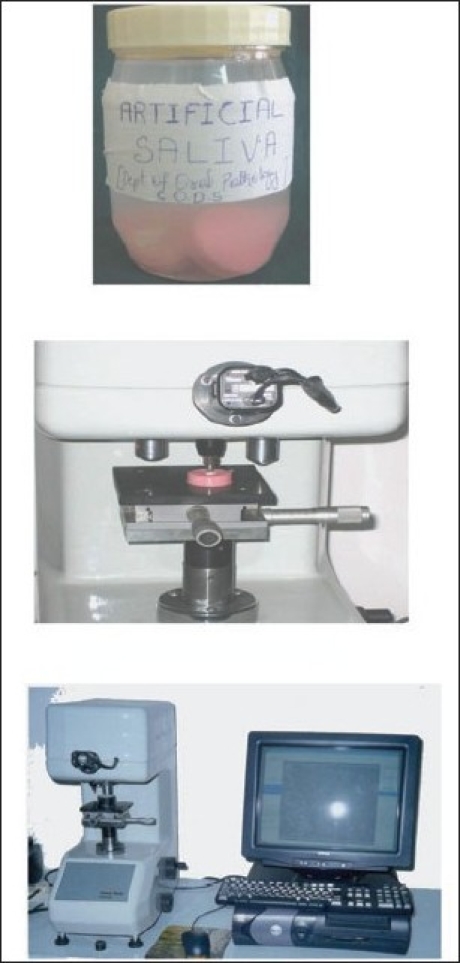
Storage and Zweck indentor

## DISCUSSION

Many patients are keeping their teeth for longer time but dislike the physiological darkening process that follows the laying down of secondary dentine.[2,15] The use of chemical agents to remove certain intrinsic stains from the enamel or the dentin of vital teeth is not new. Hydrochloric acid and hydrogen peroxide have been used alone and in combination to remove stains associated with fluorosis, tetracycline and more recently trauma related injuries.[16]

It was in the year 1966 that McInnes reported a technique that combined hydrochloric acid and hydrogen peroxide to remove fluorosis stains. He used a solution of five parts 30% hydrogen peroxide, five parts 36% hydrochloric acid and one part ethyl ether and applied the solution with a cotton wrapped toothpick to the areas of the teeth affected by the stain. After 10 to 15 minutes the teeth were washed with water and neutralized with a sodium bicarbonate paste.[17]

In the present study McInnes bleaching agent was selected, as it was most commonly used in clinical setup for treating dental fluorosis. McCoy SE[3] has reported that McInnes bleaching technique were specifically recommended for the treatment of teeth exhibiting endemic dental fluorosis because of its superficial nature, easy manipulation and its quality of being less expensive when compared to other commercially available agents like carbamide peroxide.[18] All the samples used in the study showed no change in the microhardness immediately after first cycle of bleaching. Around 0.3% reduction in the microhardness was seen which was not significant (*P* =0.034).

When the bleaching procedure was repeated after 24 hours (the second cycle of bleaching) there was significant reduction in the VHN, which shows 2.3% reduction in the microhardness, which was significant (*P* < 0.001).

In many studies[19,20] artificial saliva was used for storing the specimens in between the bleaching cycles, because it is believed that artificial saliva contributed to a slight increase in the microhardness, after demineralization.

The amount of demineralization of enamel by bleaching was assessed using a microhardness tester.[8] Microhardness test was selected mainly because it was economical compared to the other tests and was easily available (Indian Institute of Science, Bangalore).

Microhardness measurement of tooth material can be done in three different ways like; Knoop's hardness number (KHN), Vickers's hardness number (VHN) and Brennel's hardness number (BHN). In the present study Vicker hardness number was chosen over Knoop's because a square shape of indent obtained in VHN was easy and more accurate to measure. Even the minute changes in the square shape indent obtained after the test can be easily detected, where as the Knoop hardness test gave rhomboid shape indentation with opposing surfaces parallel to each other and detecting the error was difficult.

According to Chow LC, Takagi S,[21] both KHN and VHN have reported approximately the same value. The average hardness value for enamel is in the range from 270 to 350 KHN range or from 250 to 360 VHN range. In this study the average values for enamel microhardness were in the range from 266.5 VHN to 318.5 VHN range, which was within the standard range.

One of the factors that affects the hardness measurement was the specimen preparation, because any tilt or not flat surface would yield a too large an indentation and thus a smaller Vickers's hardness number. Therefore to produce a flat surface in the specimens was crucial in this study, but the cut enamel surface tested for microhardness did not have a flat surface. The convex surface gave variations in the VHN.[10] Hence two indentations were made to avoid any operational bias, then average of two indentations was taken for statistical analysis.

Various techniques have been followed to neutralize the effect of bleaching; the use of baking soda, prophylactic paste containing fluoride, APF gel and use of copious amount of water.[22] In the present study GC Tooth mousse has been used, which is a commercially available Casein phosphopeptides (CPP), stabilized amorphous calcium phosphate (ACP) product.[23]

The values obtained after the application of remineralizing solution for seven days (first cycle of remineralization) showed marginal recovery in the microhardness by 2.2% from the baseline. Although most of the remineralizing solutions were supersaturated with respect to the amorphous and crystalline calcium phosphate phases, the solutions which were stabilized by the CPP-ACP, such as in GC Tooth mousse, the spontaneous precipitation of calcium phosphate did not occur and thus the longer the duration of the solution in contact with the teeth the better was the remineralization.[9,24] The second cycle of remineralization for seven more days showed a 3% increase from the baseline value which was significant (*P* < 0.05). According to the manufactures instructions for the maximum benefit the solutions of GC Tooth mousse solution should be left on the tooth surface as long as possible. Hence in the present study the excess GC Tooth mousse was wiped off after four minutes leaving the residues on the samples and then stored in artificial saliva.

In summary, McInnes bleaching agent used in this study caused significant decrease in the enamel microhardness when it was compared with the baseline values. The more frequency of application, the lesser was the VHN. However subsequent remineralizing by GC Tooth mousse for fourteen days does cause recovery in the microhardness, which was almost equal or slightly more than the baseline value.

## CONCLUSION

From the above study it can be concluded that

- McInnes bleaching agent does decrease the microhardness of enamel by causing enamel demineralization.
- GC Tooth mousse used in the study causes an increase in the microhardness of bleached enamel by maintaining high gradient of calcium and phosphate ions at the enamel subsurface.

This study was conducted with a small number of samples utilizing *in vitro* conditions. Application to general population requires further research and analysis.
